# Supplementary material for: Cardiorespiratory Fitness and Carotid Intima–Media Thickness in Physically Active Young Adults: CHIEF Atherosclerosis Study
Source: J Clin Med. 2022 Jun 24;11(13):3653. doi: 10.3390/jcm11133653 (PMC9267611; doi:10.3390/jcm11133653)
Supplement: Supplementary file 1 [file jcm-11-03653-s001.zip › jcm-1760655-supplementary.pdf]

Supplemental Table S1. Associations between Metabolic Risk Factors and cIMT ≥0.9 mm

	Model 1		Model 2	
	OR (95% CI)	p	OR (95% CI)	p
Systolic BP ≥130 mmHg	1.100 (0.571 – 2.117)	0.77	1.001 (0.478 – 2.096)	0.99
Diastolic BP ≥85 mmHg	1.225 (0.463 – 3.243)	0.68	1.213 (0.413 – 3.561)	0.72
Hypercholesterolemia (total cholesterol ≥200 mg/dl)	1.368 (0.739 – 2.533)	0.31	1.300 (0.680 – 2.486)	0.42
Low HDL-C (<40 mg/dl for men and <50 mg/dl for women)	1.221 (0.623 – 2.390)	0.56	1.159 (0.567 – 2.367)	0.68
Triglycerides ≥150 mg/dl	1.450 (0.755 – 2.786)	0.26	1.343 (0.653 – 2.761)	0.42
Fasting glucose ≥100 mg/dl	0.786 (0.378 – 1.638)	0.52	0.752 (0.357 – 1.585)	0.45
Abdominal obesity (≥90 cm for men and ≥80 cm for women)	1.258 (0.712 – 2.225)	0.42	1.189 (0.646 – 2.189)	0.57
Hyperuricemia	0.899 (0.512 – 1.557)	0.70	0.778 (0.434 – 1.395)	0.39
Physical fitness				
Top 16% of performance level	1.000		1.000	
Middle 68% of performance level	2.113 (0.888 – 5.025)	0.09	2.044 (0.856 – 4.881)	0.10
Bottom 16% of performance level	1.766 (0.584 – 5.343)	0.31	1.608 (0.523 – 4.949)	0.40

Multiple regression analysis (Model 1) was used to determine the associations between Metabolic Risk Factors and cIMT with adjustments for age, sex, tobacco smoking and alcohol intake. Model 2 was used with adjustments for Model 1 covariates and other metabolic components.
